# Risk factors of acute and overuse musculoskeletal injuries among young conscripts: a population-based cohort study

**DOI:** 10.1186/s12891-015-0557-7

**Published:** 2015-05-01

**Authors:** Henri Taanila, Jaana H Suni, Pekka Kannus, Harri Pihlajamäki, Juha-Petri Ruohola, Jarmo Viskari, Jari Parkkari

**Affiliations:** 1 Research Department, Centre for Military Medicine, Lahti and Helsinki, Finland; 2 Tampere Research Centre of Sports Medicine, and Injury and Osteoporosis Research Center, UKK Institute, PO Box 30, FIN-33501 Tampere, Finland; 3 Research Unit of Pirkanmaa Hospital District, and Department of Orthopedics and Trauma Surgery, Tampere University Hospital, Tampere, Finland; 4 Division of Orthopedics and Traumatology, Seinäjoki Central Hospital, Seinäjoki, Finland; 5 University of Tampere, Seinäjoki, Finland; 6 Training Division of the Defence Staff, Finnish Defense Forces, Helsinki, Finland

**Keywords:** Risk factors, Sporting injuries, Physical fitness, Physical activity, Military training

## Abstract

**Background:**

Military service in Finland is compulsory for all male citizens and annually about 80% of 19-year-old men enter into the service. The elevated risk for many chronic diseases and loss of function among those who are inactive and unfit can be often detected already in youth. On the other hand, activity-induced injuries among young are true public health issue. The purpose of the present prospective cohort follow-up study was to evaluate predictive associations between acute or overuse injuries and their various intrinsic risk factors.

**Methods:**

Four successive cohorts of conscripts who formed a representative sample of Finnish young men were followed for 6 months. At the beginning of the service, the risk factors of injuries were measured and recorded and then the acute and overuse injuries treated at the garrison clinic were identified. Predictive associations between injuries and their risk factors were examined by multivariate Cox’s proportional hazard models.

**Results:**

Of the 1411 participants, 27% sustained an acute injury and 51% suffered from overuse injury. Concerning acute injuries, highest risk for severe injuries were detected among conscripts with low fitness level in both the standing long-jump and push-up tests (hazard rate, HR=5.9; 95% CI: 1.6‒21.3). A history of good degree in school sports was not a protective factor against acute injuries. High waist circumference and, on the other hand, being underweight according to BMI increased the HR for overuse injuries. Brisk leisure time physical activity before military entry was a protective factor against overuse injuries. Poor result in Cooper’s test was a warning signal of elevated risk of overuse injuries.

**Conclusion:**

We confirmed previous findings that low level of physical fitness is predictor for musculoskeletal injuries during intensive physical training. The U-shaped relationship between body composition and overuse injuries was noticed indicating that both obesity and underweight are risk factors for overuse injuries. Persons with excellent sports skills according to their earlier degrees in school sports had similar HR for acute injuries than those with poorer degrees. This indicates that school-age sports skills and fitness do not carry far and therefore preventive programmes are needed to prevent activity-induced injuries.

## Background

Numerous well-conducted studies have shown that the least active and unfit are at greatest risk for a variety of chronic diseases, loss of function, and all-cause mortality [1,2]. Western lifestyle is becoming increasingly sedentary [3], which is associated with a range of poor health outcomes, typically high levels of obesity, type 2 diabetes, cardiovascular problems [4] and mortality [5]. Moreover, there is clear evidence that low physical fitness rather than just low levels of physical activity (PA) or sedentary lifestyle, is an independent risk factor for chronic diseases, poor health outcomes [6-8] and musculoskeletal injuries [2,9,10]. The other side of the medal is that the risk of musculoskeletal injury increases with an increase in PA. In fact, the increasing number of activity-induced injuries among adolescents and young adults is becoming a true public health burden [11-13].

In military environment, previous physical inactivity [14-16] and low aerobic endurance [14,17-22] have been shown to be associated with musculoskeletal injuries during military training. Musculoskeletal injuries are the main reason for morbidity in military populations [23,24]. Moreover, training-related injuries are the main reason for disability, long-term rehabilitation, functional impairment, and premature discharges from military service [23,25-27]. In the Finnish Defence Forces, musculoskeletal injuries and disorders are the second highest reason for premature discharge from military service, and their number has been increasing [28]. Given that 80% of young men in Finland enter into military service, the high number of musculoskeletal injuries affects public health [24].

The purpose of the present 6-month prospective follow-up study of four successive cohorts conscripted in the Finnish army was to evaluate predictive associations between acute injuries (AIs) or overuse injuries (OIs) and their various intrinsic risk factors, including socio-economic, health, health behaviour, and physical fitness variables. We hypothesised that low levels of physical fitness, sedentary lifestyle and health damaging behaviour at the beginning of military service are associated with an increased incidence of acute and especially overuse injuries among young men during intensive physical training.

## Methods

### Study design

**Figure 1:**
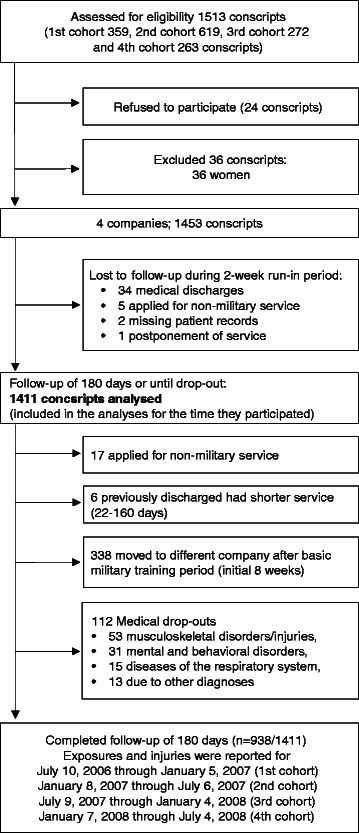
Flow of participants through the study.

In Finland, military service or alternative civil service is compulsory for all male citizens over 18 years of age and voluntary for women. Annually about 80% of 19-year-old men enter into the service. The service period varies from 6 to 12 months. The study was carried out in the Pori Brigade, a typical Finnish garrison. Companies without special qualification requirements were enrolled in the study including anti-tank, signal, mortar, and engineer companies forming a representative sample of conscripts (*N* = 1513). During the study period, four cohorts of conscripts started service in the brigade (Figure 1). Annually, the conscripts of each age-cohort were randomly assigned into the study companies. The above-described study sample was the same as in our previous study reporting predictors of military discharge [29]. Approval for the study protocol was obtained from the Ethics Committee of Pirkanmaa Hospital District (ETL-code R07076).

### Participants

The rate of written informed consent to participate was high (98%). Because there were only 36 women in the study (2.4%), their data were excluded from the analysis. Conscripts entering military service were young healthy men, all of whom had a medical check-up by a clinician during the 12 months before entering into the military. The health status of the conscripts was rechecked at baseline using routine medical screenings performed by a physician. To exclude injuries and illnesses originating before the onset of military service, conscripts discharged from the service at the medical screenings during the 2-week run-in period were excluded from the analyses leaving 1411 conscripts in the analyses. All subjects were planned to be followed for 180 days beginning on the first day of service, but some drop-out from the military or changed company and this was taken into account when calculating exposure times (Figure 1).

### Baseline characteristics of participants

**Figure 2:**
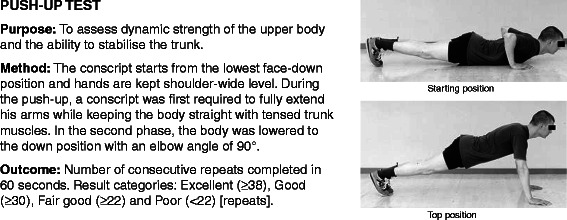
Description of push-up test.

**Figure 3:**
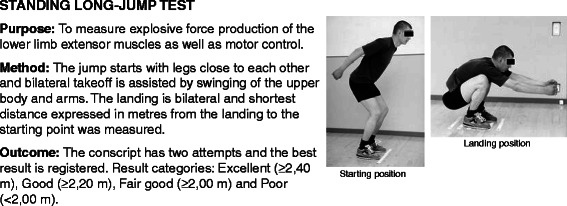
Description of standing long jump test.

**Figure 4:**
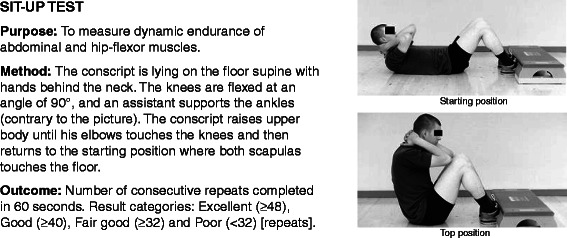
Description of sit-up test.

**Figure 5:**
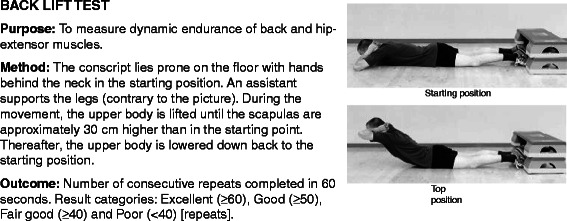
Description of back lift test.

**Figure 6:**
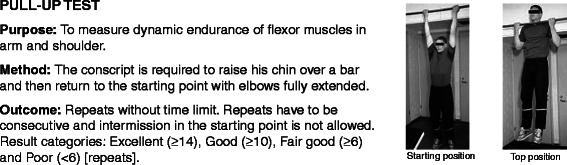
Description of pull-up test.

All conscripts filled in a standard pre-information questionnaire during the first week of military service. Questions charted conscripts’ socio-economic factors, health, and health behaviour at the baseline of the study. Baseline characteristics of the study participants are presented in Table 1. Assessment of physical fitness was conducted in 97% of the conscripts during the first 2 weeks of their service. The assessment of physical fitness and body composition were applied according to standard procedures in the Finnish Defence Forces and are reported in the previous articles [17,29] (Figures 2, 3, 4, 5 and 6; informed consent to publish was obtained from the individual in the figures). Because excellent results in the 12-min running test were uncommon (<4%), the two highest levels, good and excellent, were combined to obtain a group of equal size for comparison. Individual physical fitness test results were combined into a single variable to explore whether co-impairment in aerobic and muscular fitness would increase the HR for injuries. Co-impairment was defined as a poor result in both measured fitness tests according to the standard result categories [17,30] (Figure 2, 3, 4, 5 and 6).

**Table 1:** **Baseline characteristics of 1411 male conscripts by company**

Variable	Anti-tank	Signal	Mortar	Engineer	Missing	P-value^1^
Number of conscripts	263	540	363	245	0 (0%)	-
Age, median, years	19	19	19	19	0 (0%)	0.422 ^2^
Body mass index, median, kg/m^2^	23.4	22.6	23.3	23.6	139 (10%)	0.003 ^2^
Waist circumference, median, cm	87.0	84.9	85.6	87.0	101 (7%)	0.001 ^2^
12-minute run test result, median, m	2310	2308	2500	2400	42 (3%)	<0.001 ^2^
Conscript’s physical fitness index (CPFI)^4^, median, points	15.05	14.75	17.00	15.50	46 (3%)	<0.001 ^2^
Hometown population ≥ 10,000 persons,%	59%	66%	59%	57%	24 (2%)	0.044 ^3^
High level of education^5^,%	48%	38%	49%	35%	22 (2%)	<0.001 ^3^
High level of previous physical activity^6^,%	31%	24%	42%	36%	23 (2%)	<0.001 ^3^
Good self-assessed health^7^,%	57%	47%	61%	51%	22 (2%)	<0.001 ^3^
Chronic impairment or disability,%	17%	15%	16%	17%	27 (2%)	0.802 ^3^
Clear musculoskeletal symptoms^8^,%	28%	32%	26%	27%	23 (2%)	0.339 ^3^
Previous or current regular smoker, %	43%	47%	44%	57%	26 (2%)	0.004 ^3^
Use of alcohol ≥ 3 times per week, %	16%	19%	15%	20%	23 (2%)	0.318 ^3^

^1^P-value for difference between the companies.

^2^P-value was calculated using a Kruskal-Wallis test for median difference.

^3^P-value was calculated using χ2 statistics for difference.

^4^CPFI = (12-min running test result (metres) + 100 × muscle fitness test points) / 200, [Excellent (CPFI ≥ 21.00), Good (17.00 ≤ CPFI < 21.00), Fair good (13.00 ≤ CPFI < 17.00), Poor (CPFI < 13.00)].

^5^Graduated or studies in higher education institution.

^6^Sweating exercise at least three times per week during the last month before entering the military.

^7^Compared to age-mates.

^8^Symptoms lasting more than 7 days in at least one anatomical region during the last month before entering the military.

### Physical training programme

Conscripts performed 8 weeks of routine basic physical training programme with a gradual increase in intensity. There was an average of 17 hours of military actions per week mostly at low-to-moderate intensity including marching, cycling, skiing, orienteering, swimming, drill training and combat training. The 2-month basic training period was followed by a specific military training programme depending on the company and service duration. During this 4-month period of service, the volume and intensity of physical training was maintained at approximately the same level in different companies. Military tasks practiced in the four companies were partly different after the 2-month basic training period due to different soldiery assignment objectives depending of the company. After this, however, the core of the military physical training was still based on the same military actions as in the beginning of the service.

### Injury definition and data collection

Injury was defined as an event that resulted in physical damage or pain for which the conscript sought medical care from the garrison clinic. During military service, all conscripts had to use the services of the military healthcare units. The data were collected from July 10, 2006 to July 4, 2008 (Figure 1). The date, anatomical location, etiological circumstances, severity and diagnosis were registered in electronic patient records. Injuries that occurred during the conscript’s leisure time or on the way to vacation or back to garrison were also included in the analyses. Because the conscripts may have sought medical care several times due to the same event, the total number of health clinic visits exceeded the number of injuries. The health clinic visits were considered to be for the same injury when the conscript had sustained an injury of the same type and location during the preceding 2 weeks or if a physician had marked on the patient files that the reason for the visit was related to the previous injury.

The type of injury was categorised as acute if it had a sudden onset involving known trauma [31-33]. For example, sprains, strains, ligament ruptures, and joint dislocations were categorised as acute. Overuse-related injuries had a gradual onset without known trauma [31,33] and they were described as a pain syndrome of the musculoskeletal system, where symptoms appeared during physical activities at previously symptomless body part [34]. One of the study physicians (HT) checked all the patient records in order to differentiate acute injury (AI) from overuse injury (OI).

Injury severity was categorised according to the number of days of limited duty. Severe injury was defined as an incidence of time loss of at least 7 active service days. Limited duty involved a physical restriction that prevented the conscript from fully participating in military training events. Discharge from military service was indicated when a physician determined a conscript unable to continue military training. Discharges from military service due to musculoskeletal injuries were registered as severe injuries. Specific details of these discharges including risk factor analyses were presented previously [29].

### Statistical analysis

IBM SPSS Statistics 22.0 for Windows software was used for statistical analysis. AI and OI incidences were calculated by dividing the number of conscripts with one or more AIs or OIs treated in the garrison clinic (numerator) by the total number of conscripts (denominator) and expressed as a percentage. The incidence rates were calculated by dividing the number of injured conscripts by the exposure time and expressed per 1000 person-days. Exposure times for AI and OI incidence rates were calculated until onset of the conscript’s first AI or OI, respectively.

Cox’s proportional hazard models were applied to study the prospective associations between baseline characteristics and outcome. The primary outcomes were defined as an incidence of any AI or OI. The secondary outcomes were defined as an incidence of time loss of at least 7 active service days due to one or several acute (referred to as a severe AI) or overuse (referred to as a severe OI) injuries to explore predictors of injuries that debilitate service clearly. In the first phase of the Cox regression, each independent variable was analysed one at a time (univariate). Results are expressed as hazard ratios (HR) and calculated with 95% CIs with age at baseline forced into the model.

A multivariate Cox’s proportional hazard models (enter-method) was used to identify independent risk factors for AI and OI incidence and examine interactions between risk factors. Stepwise procedures were not used. Presenting the univariate HRs parallel to adjusted HRs was made to allow the reader to perceive how adjustments affect studied risk factors. Cox’s proportional hazard model assumptions were assessed by using the Schoenfeld residuals [35]. Conceptually compatible and logical risk factors that were possibly significant variables (*P* < 0.20) in the initial univariate models were included in the multivariate models. To exclude colinearity, we identified the variables that were highly correlated (r >0.5), and we included in the multivariate model only those variables that had a higher level of significance (e.g. waist circumference was selected in the multivariate model and BMI was not selected). Considering AIs and OIs, the included adjusting variables for multivariate models are presented in footnote of the Table 2 and Table 3, respectively. A *P* value of less than 0.05 was considered statistically significant when interpreting the results from Cox’s proportional hazard models.

**Table 2:** **Hazard ratios (HR) for any acute injury (AI) and severe acute injury (severe AI) incidence by most important socioeconomic, health and health behaviour variables at baseline**

Baseline variable	Category	Total number (% of experienced AI; % of experienced severe AI)	HR for AI incidence (n = 386)*	HR for AI incidence (n = 354)**	HR for severe AI incidence (≥7 service days lost) (n = 89)*	HR for severe AI incidence (≥7 service days lost) (n = 81)**
***Socioeconomic variables***						
Age	18–19 years	1052 (27; 6)	1 (Referent)	1 (Referent)	1 (Referent)	1 (Referent)
	20–28 years	359 (27; 6)	1.1 (0.8–1.3)	1.0 (0.8–1.3)	1.1 (0.7–1.7)	1.1 (0.7–1.8)
Father's occupation	Not physical	488 (24; 6)	1 (Referent)	1 (Referent)	1 (Referent)	1 (Referent)
	Physical	590 (29; 7)	1.3 (1.0–1.6)	1.2 (1.0–1.6)	1.0 (0.6–1.7)	1.0 (0.6–1.6)
	Unclear or unemployed	302 (28; 6)	1.2 (0.9–1.6)	1.1 (0.8–1.4)	0.9 (0.5–1.6)	0.8 (0.4–1.5)
School success (educational level and grades combined)	Excellent^1^	218 (20; 4)	1 (Referent)	1 (Referent)	1 (Referent)	1 (Referent)
	Good^2^	608 (28; 5)	**1.5 (1.1–2.1)**	**1.5 (1.1–2.1)**	1.4 (0.6–3.0)	1.5 (0.6–3.7)
	Satisfactory^3^	467 (27; 8)	**1.5 (1.1–2.1)**	1.4 (0.9–2.0)	**2.4 (1.1–5.2)**	**2.5 (1.0–6.3)**
	Poor^4^	96 (35; 13)	**2.5 (1.6–3.9)**	**2.4 (1.5–3.9)**	**4.5 (1.8–11.1)**	**5.3 (1.9–15.1)**
Urbanisation level of the place of residence	Countryside	227 (23; 5)	1 (Referent)	1 (Referent)	1 (Referent)	1 (Referent)
	Town, < 10000 inhabitants	310 (25; 8)	1.1 (0.8–1.6)	1.0 (0.7–1.4)	1.5 (0.7–3.0)	1.3 (0.6–2.5)
	City, ≥ 10000 inhabitants	552 (27; 5)	1.2 (0.9–1.7)	1.1 (0.8–1.5)	1.0 (0.5–2.0)	0.9 (0.4–1.8)
	City, ≥ 90000 inhabitants	298 (33; 8)	**1.5 (1.1–2.1)**	**1.5 (1.1–2.2)**	1.4 (0.7–2.9)	1.5 (0.7–3.0)
Company	Anti–tank company	263 (24; 6)	1 (Referent)	1 (Referent)	1 (Referent)	1 (Referent)
	Signal company	540 (27; 6)	1.3 (0.9–1.7)	1.2 (0.9–1.7)	0.9 (0.5–1.7)	1.0 (0.6–2.0)
	Mortar company	363 (21; 5)	1.2 (0.9–1.7)	1.2 (0.8–1.7)	1.2 (0.6–2.2)	1.4 (0.7–3.0)
	Engineer company	245 (40; 9)	**1.9 (1.4–2.6)**	**1.9 (1.3–2.6)**	1.5 (0.8–2.9)	1.6 (0.8–3.2)
***Health variables***						
Body mass index ^5^ (BMI = (kg) / (m)^2^)	Underweight (<18.5)	56 (21; 2)	0.9 (0.5–1.5)	0.9 (0.5–1.6)	0.3 (0.1–2.4)	0.3 (0.1–2.3)
	Normal (18.5 − 25.0)	812 (26; 6)	1 (Referent)	1 (Referent)	1 (Referent)	1 (Referent)
	Pre–obese (25.0 − 30.0)	300 (29; 7)	1.1 (0.9–1.4)	1.1 (0.8–1.4)	1.2 (0.7–2.0)	1.1 (0.6–1.8)
	Obese (≥30.0)	104 (40; 12)	**1.7 (1.2–2.3)**	1.3 (0.9–1.9)	**2.0 (1.1–3.8)**	1.2 (0.6–2.4)
Waist circumference (WC, cm)	Thin (<80 cm)	271 (25; 4)	1.0 (0.8–1.3)	1.0 (0.8–1.4)	0.8 (0.4–1.6)	0.9 (0.4–1.6)
	Average (80 – 93.5 cm)	739 (27; 6)	1 (Referent)	1 (Referent)	1 (Referent)	1 (Referent)
	Increased (94 – 101.5 cm)	178 (32; 7)	1.2 (0.9–1.7)	1.1 (0.8–1.5)	1.3 (0.7–2.4)	1.0 (0.5–1.8)
	High (≥102 cm)	122 (32; 11)	1.3 (0.9–1.9**)**	1.1 (0.8–1.7)	**2.2 (1.2–3.9)**	1.4 (0.7–2.7)
Sum factor of musculoskeletal symptoms	Minimal symptoms^6^	440 (25; 5)	1 (Referent)	1 (Referent)	1 (Referent)	1 (Referent)
	Mild symptoms^7^	548 (28; 6)	1.2 (0.9–1.5)	1.2 (0.9–1.5)	1.2 (0.7–2.0**)**	1.0 (0.6–1.7)
	Clear symptoms^8^	400 (30; 8)	**1.4 (1.0–1.8)**	1.2 (0.9–1.6)	**1.8 (1.0–3.0)**	1.3 (0.8–2.3)
Chronic disease	No	1012 (28; 6)	1 (Referent)	1 (Referent)	1 (Referent)	1 (Referent)
	Yes	377 (26; 7)	1.0 (0.8–1.2)	1.0 (0.8–1.2)	1.1 (0.7–1.8)	1.1 (0.7–1.8)
Sports injury during last month	No	1254 (27; 6)	1 (Referent)	1 (Referent)	1 (Referent)	1 (Referent)
	Yes	130 (29; 10)	1.2 (0.8–1.6)	1.2 (0.8–1.6)	**1.8 (1.0–3.3)**	**2.0 (1.1–3.6)**
***Health behaviour variables***						
Smoking habits	Never smoked regularly	735 (25; 5)	1 (Referent)	1 (Referent)	1 (Referent)	1 (Referent)
	Has smoked regularly	650 (30; 8)	**1.3 (1.1–1.6)**	1.2 (0.9–1.6)	**1.6 (1.0–2.4)**	1.2 (0.7–1.9)
Use of alcohol ^9^	<3 times per week	1148 (26; 5)	1 (Referent)	1 (Referent)	1 (Referent)	1 (Referent)
	≥3 times per week	240 (33; 12)	1.3 (1.0–1.6)	1.1 (0.8–1.4)	**2.3 (1.5–3.6)**	**2.0 (1.2–3.2)**
Drunkenness before military service	<1 time per week	1075 (26; 6)	1 (Referent)	1 (Referent)	1 (Referent)	1 (Referent)
	≥1 time per week	313 (31; 9)	**1.3 (1.0–1.6)**	1.1 (0.8–1.4)	**1.6 (1.0–2.5)**	1.1 (0.7–1.9)
Sweating exercise (Brisk leisure time sport)	≥3 times per week	438 (27; 6)	1 (Referent)	1 (Referent)	1 (Referent)	1 (Referent)
	1–2 times per week	415 (28; 5)	1.0 (0.8–1.3)	1.0 (0.7–1.3)	0.8 (0.5–1.4)	0.7 (0.4–1.2)
	Only leisured exercise	257 (27; 7)	1.1 (0.8–1.5)	0.9 (0.7–1.3)	1.2 (0.7–2.2)	0.9 (0.4–1.7)
	No physical exercise	278 (27; 7)	1.1 (0.9–1.5)	1.0 (0.7–1.4)	1.3 (0.7–2.3)	0.8 (0.4–1.6)
Belongs to a sports club	Yes, active member	206 (30; 6)	1 (Referent)	1 (Referent)	1 (Referent)	1 (Referent)
	No	1177 (27; 6)	0.9 (0.7–1.2)	0.9 (0.7–1.2)	1.1 (0.6–2.0)	1.1 (0.6–2.1)
Participates in competitive sports	Yes	180 (31; 6)	1 (Referent)	1 (Referent)	1 (Referent)	1 (Referent)
	No	1206 (27; 8)	0.9 (0.7–1.2)	0.8 (0.6–1.1)	0.8 (0.5–1.5)	0.8 (0.4–1.4)
Last degree in school sports	Good or excellent	1101 (28; 6)	1 (Referent)	1 (Referent)	1 (Referent)	1 (Referent)
	Poor or fair	286 (24; 6)	1.0 (0.8–1.3)	0.9 (0.7–1.2)	1.1 (0.7–1.9)	0.7 (0.4–1.4)

Variable distribution was charted in 1411 male conscripts during the first week of military service and discharge outcomes were registered during the following 6-month military service.

Statistically significant (p< 0.05) findings are indicated with bold type.

*Adjusted for age (univariate).

**Acute: **Adjusted for age, company, smoking (previous or current smoker), frequency of drunkenness, baseline medical conditions (sum factor of earlier musculoskeletal symptoms, sports injury during the last month before entering the military), school success (educational level and grades combined), urbanisation level of the place of residence, waist circumference and physical fitness measured as a standing long jump test result.

^1^Attended upper secondary school, polytechnic, or university and reported excellent or good grades.

^2^Other subjects from upper secondary school, polytechnic, or university and conscripts from vocational school whose grades were excellent or good.

^3^Respondents with poorer grades in vocational school.

^4^Attended only comprehensive school or had permanently interrupted vocational or upper elementary school.

^5^Not adjusted by waist circumference since BMI and WC are strongly interconnected (χ2-test, p < 0.001).

^6^‘Minimal symptoms’: maximum of 7 days in one anatomical region during the last month before military entry.

^7^'Mild symptoms’: maximum of 7 days in 2 to 6 anatomical regions during the last month before military entry.

^8^'Clear symptoms’: included the remaining conscripts i.e. those with symptoms more than 8 days or more.

^9^Not adjusted by frequency of drunkenness since drunkenness and use of alcohol are strongly interconnected (χ2-test, p < 0.001).

**Table 3:** **Hazard ratios (HR) for any overuse injury (OI) and severe overuse injury (severe OI) incidence by most important socioeconomic, health and health behaviour variables at baseline**

Baseline variable	Category	Total number (% of experienced OI; % of experienced severe OI)	HR for OI incidence (n = 721)*	HR for OI incidence (n = 634)**	HR for severe OI incidence (≥7 service days lost) (n = 251)*	HR for severe OI incidence (≥7 service days lost) (n = 212)**
***Socioeconomic variables***						
Age	18–19 years	1052 (50; 17)	1 (Referent)	1 (Referent)	1 (Referent)	1 (Referent)
	20–28 years	359 (54; 19)	1.2 (1.0–1.4)	**1.2 (1.0–1.5)**	1.2 (0.9–1.6)	1.1 (0.8–1.5)
Father's occupation	Not physical	488 (49; 16)	1 (Referent)	1 (Referent)	1 (Referent)	1 (Referent)
	Physical	590 (51; 19)	1.1 (0.9–1.3)	1.0 (0.8–1.2)	1.2 (0.9–1.6)	1.1 (0.8–1.5)
	Unclear or unemployed	302 (55; 19)	**1.3 (1.0–1.6)**	**1.3 (1.0–1.6)**	1.2 (0.8–1.7)	1.3 (0.9–1.8)
School success (educational level and grades combined)	Excellent^1^	218 (36; 10)	1 (Referent)	1 (Referent)	1 (Referent)	1 (Referent)
	Good^2^	608 (53; 18)	**1.7 (1.4–2.2)**	**1.6 (1.2–2.0)**	**1.9 (1.2–3.1)**	1.5 (0.9–2.4)
	Satisfactory^3^	467 (54; 21)	**1.9 (1.5–2.4)**	**1.4 (1.1–1.9)**	**2.4 (1.5–3.9)**	1.4 (0.9–2.4)
	Poor^4^	96 (60; 22)	**2.4 (1.7–3.4)**	**1.9 (1.3–2.7)**	**2.8 (1.5–5.1)**	1.9 (1.0–3.6)
Company	Anti–tank company	263 (52; 17)	1 (Referent)	1 (Referent)	1 (Referent)	1 (Referent)
	Signal company	540 (56; 20)	1.2 (1.0–1.4)	1.2 (0.9–1.4)	1.3 (0.9–1.8)	1.2 (0.8–1.7)
	Mortar company	363 (39; 12)	0.9 (0.7–1.1)	1.0 (0.7–1.2)	0.9 (0.6–1.4)	0.8 (0.5–1.3)
	Engineer company	245 (56; 21)	1.1 (0.9–1.4)	1.2 (0.9–1.5)	1.3 (0.9–1.9**)**	1.2 (0.8–1.9)
***Health variables***						
Body mass index^5^ (BMI = (kg)/(m)^2^)	Underweight (<18.5)	56 (61; 25)	**1.5 (1.1–2.1)**	**1.7 (1.1–2.4)**	**1.8 (1.0–3.1)**	**2.0 (1.1–3.5)**
	Normal (18.5 − 25.0)	812 (49; 16)	1 (Referent)	1 (Referent)	1 (Referent)	1 (Referent)
	Pre–obese (25.0 − 30.0)	300 (49; 13)	1.0 (0.8–1.2)	1.0 (0.8–1.2)	0.8 (0.6–1.1)	0.8 (0.5–1.1)
	Obese (≥30.0)	104 (60; 25)	**1.4 (1.1–1.9)**	1.3 (1.0–1.8)	**1.7 (1.1–2.5)**	1.3 (0.8–2.2)
Waist circumference (WC, cm)	Thin (<80 cm)	271 (48; 17)	1.0 (0.8–1.3)	1.1 (0.9–1.3)	1.2 (0.9–1.7)	1.2 (0.9–1.8)
	Average (80 – 93.5 cm)	739 (50; 16)	1 (Referent)	1 (Referent)	1 (Referent)	1 (Referent)
	Increased (94 – 101.5 cm)	178 (52; 15)	1.1 (0.9–1.4)	1.1 (0.8–1.4)	0.9 (0.6–1.4)	0.8 (0.5–1.3)
	High (≥102 cm)	122 (58; 24)	**1.4 (1.1–1.8)**	**1.5 (1.1–2.0)**	**1.7 (1.1–2.6)**	**1.7 (1.1–2.7)**
Self–assessed health^6^	Good or very good	743 (47; 14)	1 (Referent)	1 (Referent)	1 (Referent)	1 (Referent)
	Average or inferior	646 (56; 22)	**1.5 (1.3–1.7)**	1.1 (0.9–1.3)	**1.9 (1.4–2.4)**	1.2 (0.9–1.6)
Sum factor of musculoskeletal symptoms	Minimal symptoms^7^	440 (42; 10)	1 (Referent)	1 (Referent)	1 (Referent)	1 (Referent)
	Mild symptoms^8^	548 (51; 18)	**1.4 (1.2–1.7)**	**1.5 (1.2–1.9)**	**1.9 (1.3–2.8)**	**2.1 (1.4–3.2)**
	Clear symptoms^9^	400 (61; 26)	**2.0 (1.6–2.4)**	**1.9 (1.6–2.4)**	**3.1 (2.2–4.4)**	**3.3 (2.2–5.0)**
Chronic disease	No	1012 (50; 17)	1 (Referent)	1 (Referent)	1 (Referent)	1 (Referent)
	Yes	377 (53; 20)	1.1 (0.9–1.3)	1.1 (0.9–1.3)	1.2 (0.9–1.6)	1.2 (0.9–1.6)
Orthopaedic surgery	Never	1273 (50; 17)	1 (Referent)	1 (Referent)	1 (Referent)	1 (Referent)
	Yes	114 (60; 24)	1.3 (1.0–1.6)	**1.3 (1.0–1.7)**	1.4 (1.0–2.1)	1.5 (1.0–2.3)
Chronic impairment or disability^10^	No	1165 (49; 16)	1 (Referent)	1 (Referent)	1 (Referent)	1 (Referent)
	Yes	219 (60; 26)	**1.5 (1.2–1.8)**	1.2 (1.0–1.5)	**1.8 (1.4–2.4)**	**1.5 (1.1–2.1)**
Sports injury during last month	No	1254 (50; 17)	1 (Referent)	1 (Referent)	1 (Referent)	1 (Referent)
	Yes	130 (65; 23)	**1.5 (1.2–1.9)**	**1.4 (1.1–1.9)**	1.4 (1.0–2.1)	1.3 (0.8–1.9)
***Health behaviour variables***						
Smoking habits	Never smoked regularly	735 (46; 15)	1 (Referent)	1 (Referent)	1 (Referent)	1 (Referent)
	Has smoked regularly	650 (57; 21)	**1.4 (1.2–1.6)**	1.1 (0.9–1.3)	**1.6 (1.2–2.0)**	1.2 (0.9–1.6)
Use of alcohol^11^	<3 times per week	1148 (49; 17)	1 (Referent)	1 (Referent)	1 (Referent)	1 (Referent)
	≥3 times per week	240 (61; 23)	**1.3 (1.1–1.6)**	1.2 (1.0–1.4)	**1.4 (1.0–1.9)**	1.2 (0.8–1.6)
Drunkenness before military service	<1 time per week	1075 (48; 17)	1 (Referent)	1 (Referent)	1 (Referent)	1 (Referent)
	≥1 time per week	313 (60; 19)	**1.4 (1.2–1.7)**	**1.4 (1.2–1.7)**	1.2 (0.9–1.6)	1.1 (0.8–1.5)
Sweating exercise (Brisk leisure time sport)	≥3 times per week	438 (42; 13)	1 (Referent)	1 (Referent)	1 (Referent)	1 (Referent)
	1–2 times per week	415 (54; 17)	**1.4 (1.1–1.7)**	**1.2 (1.0–1.5)**	1.3 (0.9–1.9)	1.1 (0.8–1.7)
	Only leisured exercise	257 (55; 19)	**1.6 (1.3–2.1)**	**1.3 (1.0–1.7)**	**1.6 (1.1–2.4)**	1.0 (0.7–1.6)
	No physical exercise	278 (57; 26)	**1.9 (1.5–2.3)**	**1.3 (1.0–1.7)**	**2.5 (1.8–3.6)**	**1.7 (1.1–2.6)**
Belongs to a sports club	Yes, active member	206 (40; 14)	1 (Referent)	1 (Referent)	1 (Referent)	1 (Referent)
	No	1177 (53; 18)	**1.6 (1.3–2.0)**	**1.5 (1.2–2.0)**	**1.5 (1.0–2.2)**	1.2 (0.8–1.8)
Participates in competitive sports	No	1206 (51; 19)	1 (Referent)	1 (Referent)	1 (Referent)	1 (Referent)
	Yes	180 (51; 17)	0.9 (0.7–1.1)	**1.5 (1.1–2.1)**	1.0 (0.7–1.5)	**1.9 (1.2–3.0)**
Last degree in school sports	Good or excellent	1101 (49; 16)	1 (Referent)	1 (Referent)	1 (Referent)	1 (Referent)
	Poor or fair	286 (59; 23)	**1.5 (1.3–1.8)**	**1.2 (1.0–1.5)**	**1.6 (1.2–2.2)**	1.2 (0.8–1.6)

Variable distribution was charted in 1411 male conscripts during the first week of military service and discharge outcomes were registered during the following 6-month military service.

Statistically significant (p< 0.05) findings are indicated with bold type.

*Adjusted for age (univariate).

**Adjusted for age, company, smoking (previous or current smoker), frequency of drunkenness, baseline medical conditions (sum factor of earlier musculoskeletal symptoms and sports injury during the last month before military entry, previous orthopaedic operations), school success (educational level and grades combined), father’s occupational group, belonging to a sport’s club, waist circumference and physical fitness measured as a standing long jump test result.

^1^Attended upper secondary school, polytechnic, or university and reported excellent or good grades.

^2^Other subjects from upper secondary school, polytechnic, or university and conscripts from vocational school whose grades were excellent or good.

^3^Respondents with poorer grades in vocational school.

^4^Attended only comprehensive school or had interrupted vocational or upper elementary school.

^5^Not adjusted by waist circumference since BMI and WC are strongly interconnected (χ2-test, p < 0.001).

^6^Compared to age-mates.

^7^‘Minimal symptoms’: maximum of 7 days in one anatomical region during the last month before military entry.

^8^'Mild symptoms’: maximum of 7 days in 2 to 6 anatomical regions during the last month before military entry.

^9^'Clear symptoms’: included the remaining conscripts i.e. those with symptoms more than 8 days or more.

^10^Due to earlier musculoskeletal injury.

^11^Not adjusted by frequency of drunkenness since drunkenness and use of alcohol are strongly interconnected (χ2-test, p < 0.001).

## Results

During the 6-month follow-up of four successive cohorts, there were 550 acute injuries (AIs) and 1351 overuse injuries (OIs) in 1411 persons. These injuries accounted for 3435 garrison health clinic visits. Of the 1411 participants, 27% (n = 386) sustained an AI and 51% (n = 721) suffered from OI during the 6-month service. Considering severe injuries, a total of 89 (6%) conscripts suffered from severe AI, and 251 (18%) conscripts sustained a severe OI (≥7 service days lost due to injuries). The incidence rate for AI was 2.31 (95% CI: 2.09–2.55) and for OI 5.37 (95% CI: 5.00–5.78) per 1000 person-days, respectively. The AI or OI incidences for the first (AI: 29%; OI: 51%), second (AI: 28%; OI: 52%), third (AI: 25%; OI: 53%), and fourth (AI: 27%; OI: 48%) cohorts did not vary significantly (*P* > 0.20). In addition, the AI or OI incidences were not significantly different (*P* > 0.20) among arrivals entering the military in July (AI: 27%; OI: 52%) and in January (AI 27%; OI: 50%).

OIs (71%) were more than twice as prevalent as AIs (29%). Most injuries were in the lower extremities (67%) followed by the back (18%), upper extremities including shoulders (10%), head (2%) and other parts of the body (torso excluding back; 3%).

### Injury severity and associated activities

The majority (67%) of AIs were classified as minimal, leading to a maximum 3-day exemption from military training, while 18% of AIs accounted for 4–7 days , 11% for 8–28 days and 3% for over 28 days exemption from military training. Corresponding figures for OIs were 65% for a maximum 3-day exemption from military training, 20% 4–7 days, 11% for 8–28 days and 4% for over 28 days exemption from military training. Some injuries (6%) occurred during vacations and three cases (0.2%) occurred while the conscript was traveling to vacation or back to the garrison. Fifty-three (3.8%) conscripts were discharged from military service due to musculoskeletal injuries after the 2-week run-in period. Mostly (76%), these discharges were due to overuse musculoskeletal conditions (Figure 1).

### Risk factors of acute injuries

With regard to AIs, Tables 2 and 4 show the distribution and the hazard ratios of most important socioeconomic, health, health behaviour (Table 2) and physical fitness variables (Table 4) in the age-adjusted and multivariate models.

**Table 4:** **Hazard ratios (HR) for any acute injury (AI) and severe acute injury (severe AI) incidence by physical fitness variables at baseline**

Baseline variable	Category	Total number (% of experienced AI; % of experienced severe AI)	HR for AI incidence (n = 386)*	HR for AI incidence (n = 386)**	HR for severe AI incidence (≥7 service days lost) (n = 89)*	HR for severe AI incidence (≥7 service days lost) (n = 89)**
Self-assessed physical fitness^1^	Good or very good	323 (29; 6)	1 (Referent)	1 (Referent)	1 (Referent)	1 (Referent)
	Average or inferior	1066 (27; 6)	1.0 (0.8–1.3)	0.9 (0.7–1.1)	1.2 (0.7–2.0)	0.8 (0.5–1.5)
Cooper’s test (12-min running test)	Excellent (≥3000 m)	51 (22; 0)	1 (Referent)	1 (Referent)	1 (Referent)	1 (Referent)
	Good (≥2600 m)	330 (26; 5)	1 (Referent)	1 (Referent)	1 (Referent)	1 (Referent)
	Fair good (≥2200 m)	630 (28; 7)	1.2 (0.9–1.5)	1.1 (0.8–1.4)	1.6 (0.9–2.9)	1.3 (0.7–2.3)
	Poor (<2200 m)	358 (28; 7)	1.3 (1.0–1.7)	1.2 (0.9–1.7)	**1.9 (1.0–3.5)**	1.3 (0.6–2.7)
Push-up test (repeats per 60 seconds)	Excellent (≥38)	450 (26; 5)	1 (Referent)	1 (Referent)	1 (Referent)	1 (Referent)
	Good (≥30)	312 (25; 4)	0.9 (0.7–1.3)	0.9 (0.7–1.2)	0.9 (0.4–1.7)	0.7 (0.4–1.5)
	Fair good (≥22)	350 (28; 7)	1.1 (0.8–1.5)	1.0 (0.7–1.3)	1.4 (0.8–2.5)	0.9 (0.5–1.7)
	Poor (<22)	266 (32; 10)	**1.4 (1.1–1.9)**	1.2 (0.9–1.6)	**2.2 (1.3–3.9)**	1.7 (0.9–3.1)
Standing long jump test (two attempts, best result)	Excellent (≥2,40 m)	241 (22; 3)	1 (Referent)	1 (Referent)	1 (Referent)	1 (Referent)
	Good (≥2,20 m)	363 (28; 6)	1.3 (0.9–1.8)	1.2 (0.8–1.6)	1.8 (0.8–4.1)	1.6 (0.7–3.7)
	Fair good (≥2,00 m)	442 (30; 6)	1.4 (1.0–1.9)	1.2 (0.9–1.7)	1.9 (0.9–4.2)	1.7 (0.7–3.8)
	Poor (<2,00 m)	332 (28; 9)	**1.4 (1.0–2.0)**	1.2 (0.9–1.8)	**3.3 (1.5–7.1)**	**2.8 (1.2–6.4)**
Conscript’s muscle fitness index ^2^	Excellent (13–15 points)	169 (21; 4)	1 (Referent)	1 (Referent)	1 (Referent)	1 (Referent)
	Good (9–12 points)	361 (30; 6)	**1.5 (1.0–2.2)**	1.4 (1.0–2.1)	1.7 (0.7–4.3)	1.6 (0.6–4.0)
	Fair good (5–8 points)	472 (27; 6)	1.4 (0.9–2.0)	1.2 (0.8–1.8)	1.9 (0.8–4.6)	1.4 (0.6–3.6)
	Poor (0–4 points)	376 (29; 8)	**1.6 (1.1–2.4)**	1.3 (0.9–2.0)	**2.6 (1.1–6.3)**	1.8 (0.7–4.6)
Conscript’s physical fitness index^3^	Excellent (≥21,00)	69 (16; 0)	1 (Referent)	1 (Referent)	1 (Referent)	1 (Referent)
	Good (17.00 − 20.99)	409 (29; 6)	1 (Referent)	1 (Referent)	1 (Referent)	1 (Referent)
	Fair good (13.00 − 16.99)	590 (27; 7)	1.0 (0.8–1.3)	0.9 (0.7–1.1)	1.5 (0.9–2.4)	1.1 (0.6–1.9)
	Poor (<13.00)	297 (29; 8)	1.2 (0.9–1.6)	1.0 (0.7–1.4)	**1.9 (1.1–3.4)**	1.3 (0.6–2.6)
Combination of standing long jump and push–up test	Excellent^4^	144 (18; 2)	1 (Referent)	1 (Referent)	1 (Referent)	1 (Referent)
	Good^5^	447 (30; 6)	**1.7 (1.1–2.6)**	1.4 (0.9–2.2)	3.0 (0.9–9.8)	2.5 (0.8–8.4)
	Fair good^6^	650 (27; 6)	**1.6 (1.1–2.4)**	1.3 (0.8–2.0)	3.0 (0.9–9.6)	2.1 (0.6–6.9)
	Poor^7^	137 (33; 14)	**2.5 (1.5–4.0)**	**1.8 (1.0–3.0)**	**8.8 (2.6–29.8)**	**5.9 (1.6–21.3)**
Combination of standing long jump and Cooper’s test	Excellent^4^	136 (21; 2)	1 (Referent)	1 (Referent)	1 (Referent)	1 (Referent)
	Good ^5^	504 (28; 6)	1.4 (0.9–2.1)	1.4 (0.9–2.0)	3.0 (0.9–9.8)	2.7 (0.8–9.0)
	Fair good^6^	550 (27; 6)	1.4 (0.9–2.1)	1.2 (0.8–1.9)	2.8 (0.9–9.2)	2.4 (0.7–8.1)
	Poor^7^	175 (31; 12)	**1.9 (1.2–2.9)**	**1.7 (1.0–2.8)**	**7.0 (2.1–23.6)**	**5.8 (1.6–21.3)**

Variable distribution was charted in 1411 male conscripts during the first two weeks of military service and discharge outcomes were registered during the following 6-month military service.

Statistically significant (p< 0.05) findings are indicated with bold type.

*Adjusted for age (univariate).

**Adjusted for age, company, smoking (previous or current smoker), frequency of drunkenness, baseline medical conditions (sum factor of earlier musculoskeletal symptoms, sports injury during the last month before entering the military), school success (educational level and grades combined), urbanisation level of the place of residence and waist circumference.

^1^Compared to age mates.

^2^Muscle fitness index is the sum of individual muscle fitness test results including push-up, sit-up, pull-up, standing long jump and back lift tests.

^3^Conscript's physical fitness index (CPFI) = (12-min running test result (m) + 100 × muscle fitness test points) / 200.

^4^Excellent or good result in Cooper's test and excellent result in selected muscular fitness test.

^5^Excellent result in selected muscular fitness test and fair good or poor result in Cooper's test; or excellent result in Cooper's test and good, fair good, or poor result in selected muscular fitness test.

or good result in Cooper's test and good or fair good result in selected muscular fitness test; or fair good result in Cooper's test and good result in selected muscular fitness test.

^6^Poorer results than aforementioned, except the combination of poor results in both tests.

^7^Poor result in both tests.

From the *socioeconomic background* variables, a conscript’s poor school success (educational level and degrees combined) was the strongest risk factor. In multivariate analyses, poor school success was associated clearly with AI and severe AI (HR = 5.3; 95% CI: 1.9–15.1) compared to excellent school success in a graded manner. Living in bigger city with over 90000 inhabitants was associated with AI. In addition, company was associated with AI, HR being lowest in the anti-tank company and highest in the engineer company (Table 2).

With regard to *health*, pre-service musculoskeletal symptoms were associated with incidence of AI and severe AI in the age-adjusted model. After further adjustments, former sports injury (HR = 2.0; 95% CI: 1.1–3.6) remained predictive of severe AI. High BMI and high waist circumference increased the HR for severe AI in the age-adjusted model, but were not significant in multivariate model (Table 2).

With regard to *health behaviours,* health damaging behaviour was associated with incidence of AI and severe AI. Smoking and frequency of drunkenness were associated with AI in the age-adjusted model, but after final adjustments, the associations weakened (Table 2). High pre-service alcohol intake was associated with severe AI also after further adjustments in multivariate model (HR = 2.0; 95% CI: 1.2–3.2). A history of good or excellent degree in school sports was not a protective factor for AI (Table 2).

With regard to *physical fitness* single test items of poor fitness in standing long-jump and push-up test showed predictive associations with incidence AI or severe AI. Results of pull-up, back lift or sit-up tests were not associated with AIs. Association between poor push-up test and AI incidence, however, diminished after multivariable adjustments. Highest HR for both AI and severe AI were detected among conscripts with low fitness level in both standing long-jump and push-up tests (HR = 5.9; 95% CI: 1.6–21.3) or in standing long-jump and Cooper’s 12-min running test (Table 4).

### Risk factors of overuse injuries

Considering OIs, Tables 3 and 5 show the distribution and the hazard ratios of most important socioeconomic, health, health behaviour (Table 3) and physical fitness variables (Table 5) in the age-adjusted and multivariate models.

**Table 5:** **Hazard ratios (HR) for any overuse injury (OI) and severe overuse injury (severe OI) incidence by physical fitness variables at baseline**

Baseline variable	Category	Total number (% of experienced OI; % of experienced severe OI)	HR for OI incidence (n = 721)*	HR for OI incidence (n = 721)**	HR for severe OI incidence (≥7 service days lost) (n = 251)*	HR for severe OI incidence (≥7 service days lost) (n = 251)**
Self-assessed physical fitness^1^	Good or very good	323 (46; 14)	1 (Referent)	1 (Referent)	1 (Referent)	1 (Referent)
	Average or inferior	1066 (53; 19)	**1.4 (1.1–1.6)**	1.1 (0.9–1.3)	**1.5 (1.1–2.1)**	1.0 (0.7–1.5)
Cooper’s test (12-min running test)	Excellent (≥3000 m)	51 (43; 12)	1 (Referent)	1 (Referent)	1 (Referent)	1 (Referent)
	Good (≥2600 m)	330 (44; 13)	1 (Referent)	1 (Referent)	1 (Referent)	1 (Referent)
	Fair good (≥2200 m)	630 (50; 15)	**1.3 (1.1–1.5)**	1.1 (0.9–1.3)	1.2 (0.9–1.7)	1.1 (0.7–1.6)
	Poor (<2200 m)	358 (59; 26)	**1.8 (1.5–2.2)**	**1.4 (1.1–1.8)**	**2.4 (1.7–3.4)**	**1.9 (1.3–3.0)**
Push-up test (repeats per 60 seconds)	Excellent (≥38)	450 (48; 14)	1 (Referent)	1 (Referent)	1 (Referent)	1 (Referent)
	Good (≥30)	312 (52; 19)	1.2 (1.0–1.5)	1.1 (0.8–1.3)	1.3 (0.9–1.9)	1.1 (0.7–1.6)
	Fair good (≥22)	350 (49; 17)	1.1 (0.9–1.4)	1.0 (0.8–1.2)	1.3 (0.9–1.8)	0.9 (0.6–1.4)
	Poor (<22)	266 (57; 22)	**1.5 (1.2–1.8)**	1.1 (0.8–1.4)	**1.7 (1.2–2.5)**	1.0 (0.7–1.5)
Standing long jump test (two attempts, best result)	Excellent (≥2,40 m)	241 (45; 12)	1 (Referent)	1 (Referent)	1 (Referent)	1 (Referent)
	Good (≥2,20 m)	363 (50; 17)	1.2 (0.9–1.5)	1.1 (0.9–1.4)	**1.6 (1.0–2.5)**	1.5 (0.9–2.4)
	Fair good (≥2,00 m)	442 (52; 17)	1.2 (0.9–1.6)	1.1 (0.9–1.4)	1.5 (1.0–2.3)	1.4 (0.9–2.2)
	Poor (<2,00 m)	332 (56; 23)	**1.6 (1.2–2.0)**	1.2 (0.9–1.6)	**2.3 (1.5–3.6)**	**1.8 (1.1–3.0)**
Back lift test (repeats per 60 seconds)	Excellent (≥60)	660 (46; 13)	1 (Referent)	1 (Referent)	1 (Referent)	1 (Referent)
	Good (≥50)	284 (50; 22)	1.1 (0.9–1.4)	0.9 (0.8–1.2)	**1.7 (1.3–2.4)**	1.4 (1.0–2.0)
	Fair good (≥40)	291 (54; 20)	**1.3 (1.1–1.5)**	1.1 (0.9–1.3)	**1.5 (1.1–2.2)**	1.2 (0.8–1.7)
	Poor (<40)	143 (68; 25)	**1.9 (1.5–2.4)**	**1.5 (1.1–1.9)**	**2.1 (1.5–3.2)**	1.4 (0.9–2.2)
Conscript’s muscle fitness index^2^	Excellent (13–15 points)	169 (43; 13)	1 (Referent)	1 (Referent)	1 (Referent)	1 (Referent)
	Good (9–12 points)	361 (45; 13)	1.2 (0.9–1.6)	1.1 (0.9–1.5)	1.0 (0.6–1.7)	0.9 (0.6–1.6)
	Fair good (5–8 points)	472 (53; 18)	**1.5 (1.1–1.9)**	1.2 (0.9–1.6)	1.5 (1.0–2.5)	1.2 (0.7–2.0)
	Poor (0–4 points)	376 (57; 23)	**1.7 (1.3–2.3)**	1.3 (1.0–1.8)	**2.1 (1.3–3.3)**	1.3 (0.8–2.2)
Conscript’s physical fitness index^3^	Excellent (≥21,00)	69 (39; 9)	1 (Referent)	1 (Referent)	1 (Referent)	1 (Referent)
	Good (17.00 − 20.99)	409 (44; 13)	1 (Referent)	1 (Referent)	1 (Referent)	1 (Referent)
	Fair good (13.00 − 16.99)	590 (53; 17)	**1.3 (1.1–1.5)**	1.2 (1.0–1.5)	1.2 (0.9–1.7)	1.2 (0.8–1.7)
	Poor (<13.00)	297 (59; 26)	**1.8 (1.5–2.2)**	**1.4 (1.1–1.8)**	**2.4 (1.7–3.4)**	**1.7 (1.1–2.7)**
Combination of standing long jump and back lift test	Excellent^4^	177 (45; 12)	1 (Referent)	1 (Referent)	1 (Referent)	1 (Referent)
	Good^5^	523 (46; 14)	1.1 (0.8–1.4)	1.1 (0.9–1.4)	1.2 (0.7–2.0)	1.1 (0.7–1.9)
	Fair good^6^	607 (54; 21)	**1.4 (1.1–1.8)**	1.2 (1.0–1.6)	**1.9 (1.2–3.0)**	1.5 (0.9–2.5)
	Poor^7^	71 (70; 27)	**2.5 (1.7–3.5)**	**1.7 (1.2–2.6)**	**2.9 (1.6–5.4)**	1.7 (0.8–3.6)
Combination of back lift and push–up test	Excellen ^4^	335 (44; 13)	1 (Referent)	1 (Referent)	1 (Referent)	1 (Referent)
	Good^5^	440 (48; 17)	1.2 (1.0–1.5)	1.0 (0.8–1.3)	1.4 (0.9–2.0)	1.1 (0.8–1.7)
	Fair good^6^	536 (55; 20)	**1.5 (1.2–1.8)**	1.1 (0.9–1.4)	**1.7 (1.2–2.4)**	1.1 (0.7–1.7)
	Poor^7^	67 (67; 31)	**2.4 (1.7–3.4)**	**1.7 (1.2–2.5)**	**3.2 (1.9–5.5)**	1.7 (0.9–3.2)
Combination of standing long jump and Cooper’s test	Excellent^4^	136 (42; 8)	1 (Referent)	1 (Referent)	1 (Referent)	1 (Referent)
	Good^5^	504 (47; 15)	1.3 (1.0–1.7)	1.1 (0.8–1.5)	**2.1 (1.1–4.0)**	1.9 (1.0–3.6)
	Fair good^6^	550 (54; 19)	**1.6 (1.2–2.1)**	1.3 (0.9–1.7)	**2.6 (1.4–4.8)**	**2.0 (1.1–3.9)**
	Poor^7^	175 (59; 26)	**2.1 (1.5–2.9)**	**1.5 (1.0–2.2)**	**4.4 (2.2–8.4)**	**3.3 (1.6–7.0)**

Variable distribution was charted in 1411 male conscripts during the first two weeks of military service and discharge outcomes were registered during the following 6-month military service.

Statistically significant (p< 0.05) findings are indicated with bold type.

*Adjusted for age (univariate).

**Adjusted for age, company, smoking (previous or current smoker), frequency of drunkenness, baseline medical conditions (sum factor of earlier musculoskeletal symptoms and sports injury during the last month before military entry, previous orthopaedic operations), school success (educational level and grades combined), father’s occupational group, belonging to a sport’s club, waist circumference and physical fitness measured as a standing long jump test result.

^1^Compared to age mates.

^2^Muscle fitness index is the sum of individual muscle fitness test results including push-up, sit-up, pull-up, standing long jump and back lift tests.

^3^Conscript's physical fitness index (CPFI) = (12-min running test result (m) + 100 × muscle fitness test points) / 200.

^4^Excellent or good result in Cooper's test and excellent result in selected muscular fitness test.

^5^Excellent result in selected muscular fitness test and fair good or poor result in Cooper's test; or excellent result in Cooper's test and good, fair good, or poor result in selected muscular fitness test;

or good result in Cooper's test and good or fair good result in selected muscular fitness test; or fair good result in Cooper's test and good result in selected muscular fitness test.

^6^Poorer results than aforementioned, except the combination of poor results in both tests.

^7^Poor result in both tests.

Among *socioeconomic background* variables, poor school success, older age and unemployed or unclear father’s occupational status were associated with OI incidence but not with severe OI (Table 3).

Considering *health*, pre-service musculoskeletal symptoms were clearly associated with incidence of OI and severe OI (HR = 3.3; 95% CI: 2.2–5.0) in a graded manner even after further adjustments. High waist circumference (≥102 cm) and, on the other hand, being underweight (BMI < 18.5 kg/m2) increased the HR for both OI and severe OI (Table 3).

With regard to *health behaviours,* low pre-service PA remained predictive for incidence of OI and severe OI (HR = 1.7; 95% CI: 1.1–2.6) even after further adjustments including physical fitness in multivariate models. After multivariate adjustments drunkenness at least once per week before military entry was associated with OI incidence (HR = 1.4; 95% CI: 1.2–1.7). On the other hand, participation in competitive sports was also associated with OI and severe OI in multivariate models (Table 3).

Considering *physical fitness* single test items of all physical fitness tests were predictive for OI or severe OI. Associations between poor standing long-jump and poor 12-min running test results and severe OI remained predictive after multivariable adjustments. Similarly, poor results in 12-min running and back-lift test were associated with OI incidence (Table 5). Conscripts with poor level of fitness both in standing long-jump and 12-min running test had the highest HR for severe OI (HR = 3.3; 95% CI: 1.6–7.0) (Table 5).

## Discussion

Of the 1411 participants, 27% sustained an acute injury (AI) and 51% suffered from overuse injury (OI) during the 6-month service. Most injuries were in the lower extremities (67%) followed by the back (18%). Low levels of physical fitness and poor school success were associated with acute and overuse musculoskeletal injuries during military training in a graded manner. Low pre-service PA was associated with OIs but not with AIs. Interestingly, high waist circumference as a mark of abdominal obesity and low BMI, at the other extreme end, were associated with OIs indicating U-shaped relationship between OIs and body weight. Participation in competitive sports was a risk factor for OIs during military training, indicating that as the total amount of exercise increases from 17 hours per week in military setting, there is point at which OIs clearly increase.

In earlier military studies, low muscular endurance [21,22,36] and especially low aerobic endurance [14,17-22] have been shown to be predictors of injuries. In the present study, standing long-jump test performed according to the standard protocol in the Finnish Defence Forces, proved to be a good indicator for both acute and overuse injuries. The test measures explosive force production of the lower limbs and motor control ability (Figure 3) [17]. Co-impairments in cardiorespiratory and muscular fitness may be an indicator of poor ability to perform long-lasting weight bearing activities requiring both strength and aerobic capacity, such as loaded marching. Conscripts with lower cardiorespiratory fitness levels may perceive military training as more difficult and fatigue more rapidly [37]. Fatigue leads to changes in gait and kinematics in lower extremities [38,39]. This may induce musculoskeletal stress in specific body areas and predispose to injuries [39,40]. Hence, it is logical that poor results in 12-min running test predicted particularly OIs in the present study. Muscular fatigue may lead to a greater reliance on other muscle groups as the active muscle groups begin to fatigue [41]. In addition, adolescents with motor impairment have a reduced movement economy during aerobic exercise [42]. Probably, core stability as a subset of motor control [43] also has an important role in the muscular fatigue and pathogenesis of lower extremity injuries [44].

The present finding that low PA level before military entry is associated particularly with OIs is concordant with previous studies [15,16,45,46] and suggests that untrained conscripts overload their musculoskeletal structures and tissues more often than their active counterparts during military training. Physical activity may result in adaptation of the body and thereby help to prevent overuse related injuries when the conscript is subjected to new strains [18,47]. The present findings indicate that basic military training exerts a load particularly on lower limbs and low back due to high amount of weight bearing activities with backpack.

Interestingly, variables which were most strongly associated with the outcomes of the present study, namely earlier school success and physical fitness, are largely interconnected and related to PA in previous studies among the young. According to a Swedish study [48], continuation of engaging in sports exercise after childhood was clearly associated with good school success and high volume of physical exercise in childhood. In contrast to education level, poor entry-level fitness of conscripts is a modifiable predictor of injuries and amenable to prevention programmes. In our previous studies, a neuromuscular exercise and injury prevention counseling programme was effective in preventing acute ankle and upper extremity injuries [49] as well as disability due to low back pain [50] in young male army conscripts. Because even good or excellent degree in earlier school sports was not a protective factor against AIs, preventive neuromuscular interventions are needed in the whole population. Our previous studies showed that this type of intervention programme is not effective in reducing OIs [51]. Ideally, prevention programmes that would have an effect on OIs should start well before entry to military and target to increase PA while decreasing passive sitting and screen time at the same time.

It is not easy to find ways to promote PA among the young. One of the most promising ways to increase PA and decrease sedentary behaviour is cycling to school instead of passive commuting [52]. Moreover, active commuting is associated with higher levels of PA in over 27 years of follow-up, and thus, may contribute to a healthy and active lifestyle through life-course [53]. Other method proven to increase PA among young is enhancing PA during school days by encouraging to move during breaks between lessons and while commuting school travels and by adding elements to lessons that increase PA (e.g. lessons outside, more group work) [54]. It has been clearly demonstrated in various surveys that less than half [55] or recently in a objectively measured study [56] that less than 10% of elementary school children meet the PA guidelines in developed countries. Moreover, sedentary time increases and PA decreases from lower elementary school to upper elementary school [56,57]. Probably by increasing the number of school sports lessons especially in upper elementary and high school or vocational schools would have positive effect on PA. In a society level, building pedestrian walkways and cycle paths and adding PA counselling have positive results in PA at a reasonable price [58-60]. Moreover, safety issues should not be neglected with these types of programmes.

In the present study, both the high BMI and high waist circumference as a marker of obesity were associated with AI and OI in age-adjusted models. However, the associations weakened in multivariate models, but high waist circumference remained its significance for OIs. The U-shaped association by Jones et al. [61], indicating that being underweight is a risk factor also, was partly observed. Underweight conscripts according to BMI had higher HR for OIs than conscripts with normal BMI. This association was not observed among conscripts with low waist circumference. In young men low BMI may also be an indicator of low lean body mass i.e. musculature explaining the finding. The limits for underweight/thin were stricter considering BMI categories than waist circumference categories, which also probably explains the different outcomes. Furthermore, the results indicate an association between low body weight and OIs only among the most lightest (<3% of age cohort according to BMI, Table 3) group of young men. This is in consonance with previous studies reporting association between underweight and musculoskeletal injuries especially considering lower limb OIs and stress fractures during intensive military training [20,61,62]. The explanation may be that recruits with a higher BMI are able to cope better with load carriage tasks than their light counterparts [63,64], because proportional weight increase is higher among light persons.

Obesity is clearly associated with decrease in physical fitness and increased risk for musculoskeletal injuries [22,61,65] and healthcare usage [66] which leads to problems to meet military service standards [22,67]. Obesity impairs functional ability in everyday living and is associated with difficulties in physical demands with strenuous work and pain after the strain [68,69]. Obese people are impeded in sport activities, walking outdoors and up and down stairs, and in squatting, stooping and lifting [70]. High BMI alters body geometry and postural stability [71,72]. In turn, these alterations may reduce movement efficiency and increase the risk of injury [73]. Reducing weight improves the balance control in obese civilian men and decreases the risk of falling injuries [74].

Finnish compulsory military service reaching a vast majority of 19-year-old young men offers a unique opportunity for intervention against physical inactivity and obesity. Furthermore, in obese Finnish conscripts, military training assists in reducing body mass and improving cardiorespiratory fitness [75,76]. Moreover, conscripts may adopt healthy changes in nutrition and other lifestyle habits especially at the beginning of the military service [76].

The strengths of the study include the use of computerised patient files guaranteeing a consistent method for data acquisition because all patients who entered the garrison clinic were recorded. Second, the definitions of outcomes were clear and defined by ICD-10 codes set by an independent physician in the garrison clinic. Moreover, the patient files were manually checked to distinguish AIs and OIs. Third, the participation rate was high (98%). Fourth, the military environment provided highly standardised conditions for investigating the effect of individual risk factors: Conscripts underwent daily military programmes that were nearly equal considering the time, intensity and quality of physical training providing equal opportunity for food supply, rest and sleep. Fifth, the design of the study was prospective. Finally, due to compulsory nature of military service in Finland, reaching annually about 80% of the age cohort entering into the service, the population-based sample of incoming conscripts formed a comprehensive sample of Finnish young male adults who had passed their medical examination performed by a physician before military entry.

Our study has also limitations. First, although the compulsory military service concerns all Finnish male citizens, approximately 7% of all eligible men choose to perform non-military service in Finland and approximately 15% of conscripts are exempted from duty after physician examinations at the call-up or during the first week of military service due to minimum physical and mental requirements established for military service [77,78]. Second, the findings can only be generalised to young men because less than 3% of the conscripts were females and they were excluded from the analyses. Third, after the initial 8 weeks of basic training, the training programmes became more divergent due to the more specialised military tasks in each company. This also caused drop-out due to a company change (n = 338) and significant proportion (33.5%) of conscripts were not followed until the end of 180 days follow-up. On the other hand, all conscripts were followed up for the first 8 weeks of service and the results were adjusted by company. Finally, because the threshold for seeking medical care may vary between individuals, some conscripts may have been more inclined to seek professional care than others.

For the prevention of acute lower limb injuries, several practical neuromuscular training strategies are shown to be effective in team sports [32,51,79,80]. In military environment, pre-conditioning of low-fit recruits resulted in lower number of discharges and a tendency towards lower injury risk [81]. Similarly, modification of training programmes by reducing cumulative marching and by assuring enough recovery time decreased clearly OIs in Israeli and U.S. Armies [82,83]. More recently, Coppack and colleagues [84] completed a RCT-study of 1502 male and female recruits in UK. They reported that a 14-week training programme consisting of 4 warm-up exercises and 4 warm-down static stretches completed 7 times per week (total 105 minutes per week) was effective in reducing overuse anterior knee pain (adjusted HR = 0.25; 95% CI: 0.13–0.49). Another RCT-study conducted among Danish conscripts, revealed that concurrent exercise programme enhancing muscular strength, coordination, and flexibility was not effective in reducing the incidence of lower extremity OI. The intervention was speculated to be more effective in situations with a more gradual increase in load [85]. Dramatically increased number of low-fit and obese incoming conscripts during last decades forces the military training programmes to adapt to these new challenges [28,67,78]. Therefore, it has been suggested that the time frame for physical adjustment and development should be the whole duration of service. More progressive individual training programmes, coaching and goals could alleviate the problem of low-fit incoming conscripts [86].

Well-planned randomised controlled studies are needed to provide more evidence from effective interventions especially on the prevention of OIs. For example, studies investigating the effect of physical training programme in good time before entry into the compulsory military service are needed. The effect of the intervention programmes should be tested among those who are at the highest risk for musculoskeletal injuries. Moreover, more research is needed considering how positive results from evidence-based practice could be implemented into the injury prevention of everyday life.

## Conclusions

In Finland, 80% of 19-year-old men enter into the compulsory military service. Half of them suffer from OI and nearly one third sustain an AI during the 6-month military training. This study showed that a low cardiorespiratory and muscular fitness especially considering lower limb force production and motor control are associated with injuries. In addition, low pre-service PA and high waist circumference as a mark of abdominal obesity and, on the other hand, low BMI were associated with OIs indicating U-shaped relationship between OIs and body weight.

Poor entry-level cardiorespiratory and muscular fitness is a modifiable risk factor of injuries and amenable to prevention programmes. Because even good or excellent degree in earlier school sports was not a protective factor for AIs in military setting, preventive interventions are needed in all age groups. Neuromuscular training integrated to warm-up or cool-down sessions including balance and coordination exercises that enhance proprioceptive sensation may reduce the burden of acute PA related injuries in sports, in military training, in leisure time activities, as well as in school sport lessons. We suggest that in order to prevent OIs during intensive physical training more gradual onset of the training is needed among previously inactive and low-fit conscripts. A desirable goal in a pre-training programme before entering the military service could be a running distance of 2600 m or more in the 12-min running test. To distinguish young men with elevated risk on injuries before military entry, we suggest screening of all 9th grade students for low cardiorespiratory and muscular fitness.
